# Supplementary material for: Elder Mistreatment as a Risk Factor for Depression and Suicidal Ideation in Korean Older Adults
Source: Int J Environ Res Public Health. 2022 Sep 6;19(18):11165. doi: 10.3390/ijerph191811165 (PMC9517653; doi:10.3390/ijerph191811165)
Supplement: Supplementary file 1 [file ijerph-19-11165-s001.zip › ijerph-1858661-supplementary.pdf]

Table S1. Odds ratio of depression by elder mistreatment experience (Unweighted).

**Variables**	***OR***	**95% *CI***	***p*-value**
Independent variables			
EM experience			
No EM	1	(ref)	
Single type of EM	1.98	[1.66, 2.37]	< .001
Multiple types of EM	3.28	[2.33, 4.60]	< .001
Confounding variables (Covariates)			
Age (years)	.99	[.98, 1.00]	< .05
Gender			
Female	.94	[.82, 1.08]	.38
Male	1	(ref)	
Education			
< high school	1.25	[1.06, 1.47]	< .01
≥ high school	1	(ref)	
Marital status			
Married	.94	[.82, 1.07]	.34
Unmarried	1	(ref)	
Living with children			
Yes	1.38	[1.17, 1.63]	< .001
No	1	(ref)	
Area			
Rural	.83	[.74, .93]	< .01
Urban	1	(ref)	
Household income			
Quintile 1	2.69	[2.13, 3.41]	< .001
Quintile 2	2.19	[1.75, 2.75]	< .001
Quintile 3	1.67	[1.34, 2.09]	< .001
Quintile 4	1.34	[1.08, 1.66]	< .01
Quintile 5	1	(ref)	
Chronic disease			
Yes	1.53	[1.14, 2.05]	< .01
No	1	(ref)	
ADL/IADL disability			
Yes	1.84	[1.63, 2.08]	< .001
No	1	(ref)	
Cognitive impairment			
Yes	1.07	[.92, 1.25]	.35
No	1	(ref)	
Self-rated health			
Poor	4.01	[3.40, 4.72]	< .001
Good	1	(ref)	
Physical inactivity			
Yes	1.53	[1.37, 1.71]	< .001
No	1	(ref)	
Variables	*OR*	95% *CI*	*p*-value
Drinking			
Yes	.98	[.85, 1.14]	.82
No	1	(ref)	

Social participation			
Yes	.58	[.51, .65]	< .001
No	1	(ref)	
Social network	.91	[.88, .94]	< .001
Frequency of contact	.76	[.72, .80]	< .001

Note. *OR* = odds ratio; *CI* = confidence interval

Table S2. Odds ratio of suicidal ideation by elder mistreatment experience (Unweighted).

Variables	Model 1			Model 2		
	OR	95% *CI*	*p*-value	*OR*	95% *CI*	*p*-value
Independent variables						
EM experience						
No EM	1	(ref)		1	(ref)	
Single type of EM	3.25	[2.60, 4.05]	< .001	2.84	[2.26, 3.56]	< .001
Multiple types of EM	4.36	[3.06, 6.21]	< .001	3.38	[2.35, 4.87]	< .001
Confounding variables (Covariates)						
Age (years)	.94	[.92, .95]	< .001	.94	[.92, .95]	< .001
Gender						
Female	.90	[.73, 1.12]	.34	.91	[.73, 1.13]	.39
Male	1	(ref)		1	(ref)	
Education						
< high school	.94	[.74, 1.18]	.57	.89	[.70, 1.12]	.31
≥ high school	1	(ref)		1	(ref)	
Marital status						
Married	.62	[.51, .77]	< .001	.62	[.50, .76]	< .001
Unmarried	1	(ref)		1	(ref)	
Living with children						
Yes	1.07	[.83, 1.39]	.59	.99	[.76, 1.28]	.92
No	1	(ref)		1	(ref)	
Area						
Rural	.88	[.73, 1.06]	.19	.93	[.77, 1.13]	.48
Urban	1	(ref)		1	(ref)	
Household income						
Quintile 1	1.75	[1.22, 2.50]	< .01	1.39	[.97, 2.00]	.08
Quintile 2	1.47	[1.04, 2.08]	< .05	1.23	[.87, 1.75]	.24
Quintile 3	1.35	[.97, 1.88]	.08	1.20	[.86, 1.68]	.29
Quintile 4	1.13	[.82, 1.57]	.46	1.06	[.76, 1.48]	.72
Quintile 5	1	(ref)		1	(ref)	
Chronic disease						
Yes	1.58	[1.03, 2.43]	< .05	1.47	[.95, 2.28]	.08
No	1	(ref)		1	(ref)	
ADL/IADL disability						
Yes	1.34	[1.10, 1.63]	< .01	1.14	[.93, 1.40]	.22
No	1	(ref)		1	(ref)	
Cognitive impairment						
Yes	1.22	[.98, 1.53]	.08	1.19	[.95, 1.50]	.13
No	1	(ref)		1	(ref)	
Self-rated health						
Poor	2.20	[1.74, 2.79]	< .001	1.66	[1.30, 2.13]	< .001
Good	1	(ref)		1	(ref)	
Physical inactivity						
Yes	1.02	[.85, 1.22]	.87	.88	[.73, 1.06]	.18
No	1	(ref)		1	(ref)	
Drinking						
Yes	1.16	[.93, 1.43]	.19	1.17	[.94, 1.46]	.16
No	1	(ref)		1	(ref)	

Social participation						
Yes	.73	[.60, .89]	< .01	.86	[.70, 1.05]	.13
No	1	(ref)		1	(ref)	
Social network	1.03	[1.00, 1.07]	.08	1.05	[1.02, 1.09]	< .01
Frequency of contact	.83	[.76, .91]	< .001	.90	[.83, .99]	< .05
Depression						
Yes				4.35	[3.58, 5.28]	< .001
No				1	(ref)	

Note. *OR* = odds ratio; *CI* = confidence interval
